# Supplementary Effect of Choline Alfoscerate on Speech Recognition in Patients With Age-Related Hearing Loss: A Prospective Study in 34 Patients (57 Ears)

**DOI:** 10.3389/fnagi.2021.684519

**Published:** 2021-06-04

**Authors:** Gina Na, Sang Hyun Kwak, Seung Hyun Jang, Hye Eun Noh, Jungghi Kim, SeungJoon Yang, Jinsei Jung

**Affiliations:** ^1^Department of Otorhinolaryngology, Yonsei University College of Medicine, Seoul, South Korea; ^2^Department of Otorhinolaryngology, Ilsan Paik Hospital, Inje University College of Medicine, Goyang, South Korea; ^3^Department of Otorhinolaryngology, St. Vincent Hospital, College of Medicine, The Catholic University of Korea, Seoul, South Korea

**Keywords:** age-related hearing loss, hearing aids, choline alfoscerate, abbreviated profile of hearing aid benefit, listening comprehension

## Abstract

To investigate the effect of choline alfoscerate (CA) on hearing amplification in patients with age related hearing loss, we performed a prospective case-control observational study from March 2016 to September 2020. We assessed patients with bilateral word recognition score (WRS) <50% using monosyllabic words. The patients were 65–85 years old, without any history of dementia, Alzheimer’s disease, parkinsonism, or depression. After enrollment, all patients started using hearing aids (HA). The CA group received a daily dose of 800 mg CA for 11 months. We performed between-group comparisons of audiological data, including pure tone audiometry, WRS, HA fitting data obtained using real-ear measurement (REM), and the Abbreviated Profile of Hearing Aid benefit scores after treatment. After CA administration, the WRS improved significantly in the CA group (4.2 ± 8.3%), but deteriorated in the control group (−0.6 ± 8.1%, *p* = 0.035). However, there was no significant between-group difference in the change in pure tone thresholds and aided speech intelligibility index calculated from REM. These findings suggest that the difference in WRS was relevant to central speech understanding rather than peripheral audibility. Therefore, administering oral CA could effectively enrich listening comprehension in older HA users.

## Introduction

Age-related hearing loss (ARHL) or presbycusis is one of the most common neurodegenerative ailments in developed countries. It causes distorted communication and also impedes older adults from participating in psychosocial activities due to social isolation, loss of self-esteem, and depression (Disease et al., 2016). Moreover, hearing impairment is considered a modifiable risk factor for dementia prevention (Livingston et al., 2020). As there is no cure for presbycusis, hearing aids (HAs) have been strongly recommended to allow enriched audibility, ease of communication (EC), and cognitive decline prevention in older adults (Livingston et al., 2020). In the aging population, hearing difficulty is attributed to peripheral hearing loss associated with cochlear degeneration; central auditory processing deficit from the cochlear nucleus to the primary auditory cortex; or cognitive decline in various domains, including executive function, language memory, situational and semantic long-term memory, and psychomotor processing (Humes et al., 2012; Ronnberg et al., 2013; Panza et al., 2015). Although several studies have reported cognitive-sensory interactions, including the association between working memory and speech perception through diverse tests, multifactorial conditions including age and/or auditory system pathology may affect ARHL. Moreover, there is no evidence regarding the apparent direction of causality (Akeroyd, 2008; Humes et al., 2012; Murphy et al., 2018).

From an audiological perspective, hearing loss, which is clinically distinctive for high-frequency hearing loss and the roll-over phenomenon, results in impaired hearing sensitivity and recognition, particularly with respect to noise, central sound processing, and sound localization (Gates and Mills, 2005). Pathologic and neuroanatomical changes in ARHL include cell degeneration in the cochlea and auditory nerve, as well as cortical neuroplasticity changes, including decreased gray matter volume in the auditory cortex, anterior cingulate, and superior and medial frontal gyri (Gates and Mills, 2005; Husain et al., 2011; Eckert et al., 2012). Furthermore, compared with participants with normal hearing, patients with ARHL present with a greater decrease in spontaneous activity and local connectivity in the parahippocampal gyri and hippocampus on functional magnetic resonance imaging (MRI) (Chen et al., 2018, 2020). Therefore, improving audibility through acoustic amplification or cochlear implantation with prolonged auditory deprivation allows limited gains in speech intelligibility among older adults with ARHL (Lazard et al., 2012).

Choline alfoscerate (CA, L-alpha-glycerylphosphorylcholine), a semisynthetic derivative of phosphatidylcholine, is a common acetylcholine precursor in the brain (Amenta et al., 2001). CA reportedly enhances memory and cognition in patients with Alzheimer’s disease (AD) and stroke (Lee S.H. et al., 2017). Moreover, its neuroprotective effects have been reported in experimental models of AD, stroke, and pilocarpine-induced seizures (Lee S.H. et al., 2017; Catanesi et al., 2020). For these neurodegenerative diseases, the use of nutraceuticals for cholinergic neurotransmission could be a treatment option; further, several studies have reported the effect of CA on cognitive improvement (De Jesus Moreno Moreno, 2003; Lee S.H. et al., 2017). Considering cholinergic signaling in thalamocortical neurons of the medial geniculate body (MGB), which play a core role as major synaptic stations in central auditory processing, as well as the association between ARHL and cognition decline, we hypothesized that CA would contribute toward improving speech recognition in patients with ARHL. We aimed to assess the effect of oral CA on speech detection and recognition among HA users with ARHL.

## Materials and Methods

### Ethics Approval

This study was approved by the Institutional Review Board (IRB) of Severance Hospital (4-2017 1152) and conducted following the principles of the Declaration of Helsinki. All research was performed in accordance with relevant guidelines and regulations from the IRB of Severance Hospital. All participants signed an informed consent.

### Prospective Study Design

**FIGURE 1:**
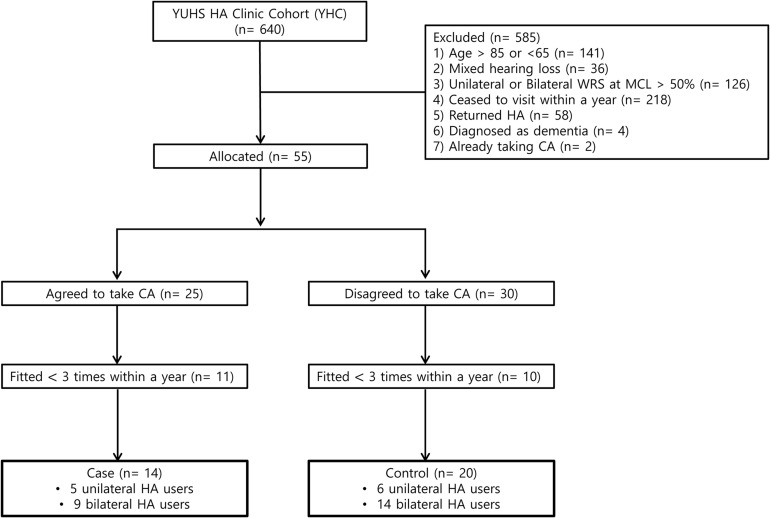
Flow chart of the case-control study for evaluating the effect of oral choline alfoscerate on speech detection and recognition in hearing aid users with aging-related hearing loss.

We analyzed 640 patients who attended the Yonsei University Health System Hearing Aids Clinic (YHC) between March 2016 and September 2020. The inclusion criteria were as follows: (1) aged >65 and <85 years; (2) sensorineural hearing loss; (3) <50% of the bilateral word recognition score (WRS) at the most comfortable loudness level (MCL) with unaided ears; (4) received newly prescribed HA from an otologist (JJ); (5) completion of 1-year follow-up; (6) HA fitting over three times within a year; and (7) being a literate, native Korean speaker. The exclusion criteria were as follows: (1) previous diagnosis of dementia, AD, parkinsonism, and depression; (2) taking CA previously prescribed by other clinicians; and (3) >90% compliance with CA administration determined by assessing the remaining pills at every visit. After 1 month of HA use, all patients received information on the original indication for the use of CA and its unknown effects on listening comprehension, and were asked whether they agreed to take CA. Subsequently, the patients were allocated to the CA or control group according to their decision. By means of the inclusion and exclusion criteria, 606 patients were excluded. Finally, 23 ears of 14 patients in the CA group and 34 ears of 20 patients in the control group were comprehensively analyzed (Figure 1). The patients in the CA group took medicine for 11 months, while they used HA. In contrast, the patients in the control group only used HA. Consequently, primary outcomes were evaluated 1 year after initial fitting of HA.

### Audiological Data

Audiological evaluation was performed as previously described (Jung et al., 2016, 2018; Lee H.J. et al., 2017). Baseline hearing sensitivity was measured using pure tone audiometry (PTA) with insert earphones or supra-aural headphones in a double-walled sound-treated booth by experienced audiologists. The aided sound-field threshold was measured with the patient sitting 1 meter away from two loudspeakers at a 45° angle. The average threshold (PTA_4_) was calculated using the thresholds at 500, 1000, 2000, and 4000 Hz; moreover, we calculated the average threshold of high frequencies (PTA_3_) at 1000, 2000, and 4000 Hz. Baseline and aided word recognition tests in a quiet environment were performed under similar conditions as PTA. We measured the WRS (%) at the MCL using 50 monosyllabic words from Hahm’s list, which comprised phonetically balanced Korean standard words (Han and Park, 1981). We defined MCL as the hearing level at which speaking was most comfortably heard. Non-tested ears were masked using a calibrated headphone (TDH-39).

### HA Fitting Data

The digital, wide dynamic range compression HA and the NAL-NL2 fitting program were prescribed for each patient as previously described (Lee et al., 2020). Probe microphone measurement was performed using the Otometrics Aurical FreeFit and Probe Microphone Measurements (PMM) module (Taastrup, Denmark) with the International Speech Test Signal (ISTS) at 55 dB (soft speech) and 65 dB sound pressure level (SPL) (conversational level speech) using two external speakers for HA verification. We calculated the proximity from the prescriptive fitting target based on the baseline PTA to the rear-ear aided response (REAR) using the root-mean-square error (RMSE) of 500, 1000, 2000, and 4000 Hz.

RMSE=14∑(Targetf-REARf)2,f=500, 1000, 2000,and 4000Hz

RMSE changes between 1 month and 1 year after wearing the HA were compared to confirm between-group uniformity of HA fitting. There was no significant difference in the HA output (REAR) between 1 month and 1 year after amplification (Supplementary Table 1). Additionally, there were stable changes in RMSE from the HA prescriptive targets at soft speech and conversational level speech (Supplementary Figure 1).

### Aided Speech Audibility

The speech intelligibility index (SII) is a calculated value for determining whether sound energy sufficient for audibility is transmitted based on each frequency, particularly weighting mid-frequencies important for speech listening. The aided SII was automatically calculated with the Aurical FreeFit/PMM system using the ISTS as defined by ANSI S3.5-1997 (R2002) (American National Standards Institute, 1997). This calculation was applied to third-octave bands with consideration of the insertion gain at 65 dB SPL (conversational level speech).

### Abbreviated Profile of Hearing Aid Benefit

The Korean version of abbreviated profile of hearing aid benefit (APHAB) was used to quantify hearing disability at 1 month and 1 year after HA fitting. The APHAB comprises 24 items scored in four subscales: EC, reverberation (RV), background noise (BN), and aversiveness of sound (AV) (Cox and Alexander, 1995). The patients were asked questions regarding various situations of hearing aid usage and frequency in daily life. Subscales of the questionnaire were assessed separately, as EC, RV, and BN reflect the listening experience under various environmental conditions, while AV reflects the discomfort caused by environmental noise; the global score, is an average of EC, RV, and BN, and AV scores (Johnson et al., 2010).

### HA Experience and Usage

Patients who had previously used HA before the HA fitting at YHC were examined before HA prescription. We found that 30.4 and 29.4% of the ears in the CA and control group, respectively, had previously used HA before the enrollment but were non-users due to dissatisfaction. The daily hours of HA usage were classified using self-reported data: <1, 1–4, 4–8, and 8–16 h at every visit. Moreover, 91.3 and 88.2% of ears in the CA and control group, respectively, were amplified for >4 h a day.

### Statistical Analyses

All statistical analyses were completed using GraphPad Prism 8 (San Diego, CA, United States). Independent *t*-test, Mann–Whitney *U*-test, and chi-square test were used for between-group comparisons of quantitative variables, including audiometric data, HA fitting data, and questionnaires. The correlation between changes in the WRS and SII was analyzed using the Pearson correlation test. Statistical significance was set at *p* < 0.05.

## Results

We analyzed 23 ears (14 patients) and 34 ears (20 patients) in the CA and control groups, respectively. Patients in the CA group received a daily oral dose of 800 mg CA for 11 months. Compliance with treatment was assessed at every visit. Participant mean [SD] age in the CA and control groups was 74.13 [6.12] and 75.21 [5.21] years, respectively (Table 1). In both groups, various HA types were used according to receiver preference, with the in-ear type being the most commonly used type. Patients were followed up for 1 year with visits at the 1st, 3rd, 6, and 12th months from the initial day of HA fitting. Additionally, aided PTA, WRS at the MCL, SII calculation from rear-ear measurements and probe microphone measurement for speech mapping were performed at each visit.

**TABLE 1:** Patient characteristics.

	CA group (23 ears, *n* = 14)	Control group (34 ears, *n* = 20)	*p*-Value
Age, mean [SD], year^†^	74.1 [6.1]	75.2 [5.2]	0.48
Sex^‡^			0.22
Male	12 ears, *n* = 8	22 ears, *n* = 13	
Female	11 ears, *n* = 6	12 ears, *n* = 7	
HA experience, ears ^‡^	7 (30.4%)	10 (29.4%)	0.93
HA side, *n*^‡^			>0.99
Unilateral	5 (35.7%)	6 (30%)	
Bilateral	9 (64.3%)	14 (70%)	
HA type, ears^‡^			0.37
BTE	0 (0%)	2 (5.9%)	
RIC	14 (60.9%)	18 (52.9%)	
CIC	8 (34.8%)	14 (41.2%)	
ITC	1 (4.3%)	0 (0%)	
HA usage hours/day, ears^‡^			0.37
1 ≤ < 4	2 (8.7%)	4 (11.8%)	
4 ≤ < 8	11 (47.8%)	10 (29.4%)	
8 ≤ < 16	10 (43.5%)	20 (58.8%)	
Medical history, ears			
Hypertension	8 (34.8%)	4 (11.8%)	
Diabetes	6 (26.1%)	3 (8.8%)	
Cardiovascular disease	4 (17.4%)	1 (2.9%)	

*BTE, behind the ear; CA, choline alfoscerate; CIC, completely in-canal; HA, hearing aid; ITC, in the canal; RIC, receiver in canal. ^†^Independent t-test. ^‡^Chi-square test.*

### Initial Assessment

There was no significant difference between the CA group and control group in the baseline pure tone average threshold at 500, 1000, 2000, 4000 Hz (PTA_4_) (59.57 [8.97] and 60.26 [12.64] dB HL), PTA_3_ (average threshold at 1000, 2000, and 4000 Hz) (62.61 [9.92] and 64.26 [12.60] dB HL), and WRS at MCL (29.83 [12.56] and 34.35 [11.20] %) (*p* = 0.82, 0.60, and 0.16, respectively) (Table 2 and Supplementary Figure 2A). Supplementary Figure 2B presents the distribution of the baseline WRS as a function of PTA_3_, which is the average threshold of high frequencies (Yellin et al., 1989; Gates et al., 2003). The dotted line was adopted from the report by Yellin et al. (1989) representing the estimated norm of cochlear ARHL for differentiation from retro-cochlear ARHL. There were 9 (39.1%) and 4 (11.8%) ears in the CA and control groups, respectively, beneath the estimated threshold of hearing loss possibly resulting from the retro-cochlear lesion.

**TABLE 2:** Audiological evaluations.

	CA group	Control group		
			
Baseline	23 ears, *n* = 14	34 ears, *n* = 20	*p*-Value^†^	
PTA_4_, mean [SD], dBHL	59.6 [9.0]	60.3 [12.6]	0.82	
PTA_3_, mean [SD], dBHL	62.6 [9.9]	64.3 [12.6]	0.60	
WRS at MCL, mean [SD], %	29.8 [12.6]	34.4 [11.2]	0.16	
MCL, mean [SD], dBHL	79.5 [7.3]	81.0 [8.9]	0.50	
**1 month**	**23 ears, *n* = 14**	**34 ears, *n* = 20**	***p*-value^†^**	
Aided PTA average, mean [SD], dBHL	50.4 [6.3]	47.4 [9.8]	0.20	
Aided WRS at MCL, mean [SD], %	38.3 [15.2]	49.0 [14.7]	0.01^∗^	
Aided MCL, mean [SD], dBHL	69.0 [3.9]	68.2 [4.8]	0.56	
Aided SII at 65 dB SPL, mean [SD], %	29.8 [12.6]	39.3 [16.0]	0.03^∗^	
APHAB	**12 ears, *n* = 7**	**28 ears, *n* = 16**	***U*-value^‡^**	***p*-value^‡^**
Global, median [IQRs]	64 [41.55–69.33]	55.5 [52.78–64.7]	135	0.34
Aversiveness, median [IQRs]	50 [8.25–62]	48 [29–78.5]	152	0.65
**1 year**	**23 ears, *n* = 14**	**34 ears, *n* = 20**	***p*-value^†^**	
Aided PTA average, mean [SD], dBHL	47.6 [5.9]	48.1 [8.4]	0.83	
Aided WRS at MCL, mean [SD], %	42.4 [16.9]	48.4 [15.7]	0.18	
Aided MCL, mean [SD], dBHL	67.7 [4.3]	66.8 [6.0]	0.53	
Aided SII at 65dBSPL, mean [SD], %	35.7 [13.6]	37.6 [13.2]	0.59	
APHAB	**17 ears, *n* = 10**	**26 ears, *n* = 15**	***U*-value^‡^**	***p*-value^‡^**
Global, median [IQRs]	59 [43.5–73.85]	52 [34–67.48]	156	0.11
Aversiveness, median [IQRs]	52 [36.5–63.5]	36.5 [17.25–73]	213	0.85

*APHAB, abbreviated profile of hearing aid benefit; CA, choline alfoscerate; MCL, most comfort loudness; PTA_3_, the average pure tone threshold at 1000, 2000, and 4000 Hz; PTA_4_, the average pure tone threshold at 500, 1000, 2000, and 4000 Hz; SII, speech intelligibility index; WRS, Word Recognition Score. ^†^Independent t-test. ^‡^Mann–Whitney U-test.*

After 1 month of HA usage, there was no between-group difference in the aided PTA_4_ as a frequency function (*p* = 0.20). However, aided WRS at MCL was significantly higher in the control group (49 [14.72] %) than in the CA group (38.26 [15.21] %) (*p* = 0.01). Furthermore, although the number of control ears was lower, aided SII was significantly higher in the control group (39.29 ± 16.05%) than in the CA group (29.83 [12.56] %) (*p* = 0.03). There was no significant between-group difference in both the APHAB scores (Global and Aversiveness scores) (Supplementary Figure 3, *p* = 0.34 and 0.65, respectively).

### Changes of Audiological Outcome After 1-Year Usage of HA

After 1 year of HA usage, there was no significant difference between the CA group and control group in aided PTA_4_ (47.61 [5.92] and 48.05[8.43] dB HL), aided WRS (42.43 [16.94] and 48.41 [15.68] %), and aided SII (35.65 [13.59] and 37.64 [13.25] %) (*p* = 0.83, 0.18, and 0.59, respectively). Further, there was no significant between-group difference in the APHAB score (Table 2).

**FIGURE 2:**
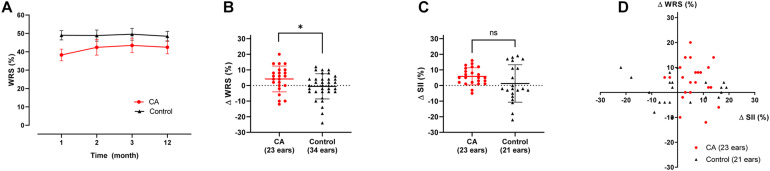
Changes in audiological outcomes at 1 year after HA use. **(A)** Aided WRS as a function of wearing duration. Although the control group showed a stable aided WRS, the choline alfoscerate (CA) group showed a rising tendency in the WRS. **(B)** Changes in the aided WRS between 1 month and 1 year after HA wearing at each group. Notably, following 1 year of CA administration, the aided WRS significantly improved in the CA group but deteriorated in the control group (*p* = 0.035). **(C)** There was no significant between-group difference in the change in the aided SII between 1 month and 1 year after using HA (*p* = 0.110). **(D)** There was no significant correlation between changes in the aided WRS and SII (the CA group: *p* = 0.930; the control group: *p* = 0.660).

Regarding the 1-year changes in the aided WRS, compared with the control group, the CA group revealed a trend toward improvement (Figure 2A). Notably, after 1 year of CA administration, the aided WRS had significantly improved (4.17 [8.29] %) in the CA group and deteriorated (−0.59 [8.07] %) in the control group (Figure 2B, *p* = 0.035). Contrastingly, there was no between-group difference in the change of aided SII (Figure 2C, *p* = 0.11). There was no significant correlation between changes in the WRS and SII (Figure 2D, CA group: *p* = 0.93; control group: *p* = 0.66). This suggests that improved speech intelligibility in the CA group is attributable to central cognitive function, with respect to speech comprehension, rather than peripheral audibility.

## Discussion

This study aimed to evaluate whether CA administration plays an adjuvant role in amplification for auditory perception, including audibility and speech discrimination, in older adults who are HA users with a WRS of <50%. We analyzed several audiological measurements—PTA_4_, WRS, SII, and APHAB score—to determine the parameters affected by CA during auditory processing. Change in the WRS was the only salient auditory function index across a year. CA administration did not improve audibility reflected by SII; however, it significantly improved speech discrimination. Therefore, we consider that CA contributes to improved speech intelligibility, which is irrelevant to peripheral audibility; rather, it involves the central cognitive ability, including speech comprehension, which occurs in parts other than the peripheral auditory organ.

Given the relatively low feasibility of amplification in individuals with ARHL whose speech discrimination is <50% (amplification had an insufficient benefit other than lip-reading or noticing environmental sounds due to poor WRS), studies on amplification in this context are scarce. Moreover, few studies have reported the speech discrimination score and fitting data in the older-adult population (Dillon, 2012; Kim et al., 2020). Pedersen et al. reported a gradual decrease in the speech discrimination score by 8−10% after 11 years in an older-adult population in their 70s (Pedersen et al., 1991). Dubno (2015) reported a similar tendency showing a gradual decline of speech discrimination in older adults, which accelerated near the age of 75 to 80 years. This is consistent with our findings of a relative decrease in the aided WRS in our control group.

ARHL results from several related deteriorations, including peripheral and central auditory processing and cognition. The primary, non-invasive step for addressing ARHL is amplification to complement the peripheral auditory processing. HA use may reduce the listening effort; however, in severe hearing loss, it cannot normalize the temporal and frequency resolution (Ronnberg et al., 2013). Furthermore, in cases of cochlear dead region, off-frequency listening causes distorted sound and impedes optimal amplification. Therefore, there is a need for an effective supplementary strategy to improve aural rehabilitation. However, fitting HAs and obtaining an audiological gain is very challenging among patients with WRS <50%. Given the negative correlation between satisfaction with HAs and the initial WRS, there is a need to improve speech intelligibility and audibility through HAs. Unfortunately, there remains no option for improving speech intelligibility. Therefore, the CA effect on WRS is very promising for improving satisfaction with and decreasing the rejection rate of HAs.

Numerous studies have reported the role of the cholinergic system in the brain; specifically, in memory and cognitive function, which are significantly degraded in aging-related dementia and AD (Davies and Maloney, 1976; Grothe et al., 2014; Haam and Yakel, 2017). In these diseases, CA directly increases cholinergic transmission as an acetylcholine precursor in the hippocampus to ameliorate cognitive symptoms or exerts neuroprotective effects by activating the neurotrophin survival pathway (Ciriaco et al., 1992; Sigala et al., 1992; De Jesus Moreno Moreno, 2003). Accordingly, several studies have examined the relationship between ARHL and the cholinergic system. Acetylcholine is an efferent neurotransmitter in the medial olivocochlear nucleus that is secreted to outer hair cells (OHC) for efferent inhibition. The resulting OHC hyperpolarization allows discrimination of sounds in BN and separation of the frequency resolution (Winslow and Sachs, 1988; Glowatzki and Fuchs, 2000). Tang et al. (2014) reported a decrease in mRNA and protein expression of the nicotinic acetylcholine receptor (nAChR) subunit β2 in the spiral ganglion neurons of old mice (24−32 months) compared with that in young adult mice (2−3 months). Further, given the higher level of central auditory processing, the MGB in the auditory thalamus codes, gates, and relays auditory information to the auditory cortex (AC) and limbic structures (Guillery et al., 1998; Richardson et al., 2020). In rats, there is a conspicuous decrease in mRNA levels of nAChR subunits with aging compared with other brain regions with the highest expression levels reported in young rats (7–14 months of age) (Ferrari et al., 1999). The MGB receives descending signals from the AC (excitatory input) and the thalamic reticular nucleus (inhibitory input). Moreover, ascending signals from the inferior colliculus (IC), both excitatory and inhibitory sensory inputs, modulate the MGB. In thalamocortical neurons in the MGB, acetylcholine has both presynaptic and postsynaptic functions. Cholinergic input from the pedunculopontine tegmental nuclei regulates MGB neuronal activity directly (via somatodendritic AChR) or indirectly (via presynaptic AChR by GABA modulation or glutamate release) (Richardson et al., 2020). Sottile et al. reported that tectothalamic inhibitory projections from the IC reached the MGB through presynaptic cholinergic input and were affected by the aging-induced decrease in nAChR activation in rats (Sottile et al., 2017b). Therefore, aging might result in deteriorated signal-to-noise ratio and sound detection (Sottile et al., 2017a). Additionally, aging may reduce nAChR density in the MGB, which evokes postsynaptic excitation and contributes to loss of speech comprehension in older adults (Sottile et al., 2017b).

Moreover, cholinergic dysfunction affects the hippocampus and modulates memory function (Haam and Yakel, 2017). The hippocampus, which is the limbic system core and is involved in memory formation and sensory processing including auditory information (Diederen et al., 2010), is part of a system for auditory working memory that maintains sounds in memory (Kumar et al., 2016). Moreover, voxel-based MRI analysis has revealed distinct hippocampal atrophy in older adults with ARHL compared with those showing mild-to-normal hearing (Aoki et al., 2020). Ruan et al. (2018) reported that withdrawing the cholinergic input to neuropeptide neuroglia from hippocampal cells, mainly under cholinergic regulation, may be involved in the excitation-inhibition imbalance in the central auditory system by affecting GABA release. Although we did not elucidate the mechanism underlying CA-mediated amelioration of deteriorated speech discrimination, CA could supply acetylcholine as a choline donor in the hippocampus. Moreover, we can speculate the benefit of CA in ARHL as a supplementary rehabilitation.

This study has several limitations. First, to explore the effect of CA on the entire auditory processing, using peripheral auditory tests in a quiet environment is insufficient and further experimental tools for verifying central auditory processing, working memory, or long-term memory are required. Second, the sample size was too small to make a definitive conclusion and more extensive studies are warranted to confirm these findings. Third, given that the study was not a randomized controlled neither blinded study, individuals who agreed to participate in the supplementary rehabilitation were more motivated and cooperative, resulting in selection bias. Fourth, even though the distribution of medical histories such as hypertension, diabetes, and cardiovascular disease was inconsistent in two groups, we could not reach their complete medical record. The severity of the disease or the effect of other medication they were taking could have affected their hearing progression. Fifth, the side effect of CA has not yet been revealed in the clinical study dealing with patients without cognitive disorders (Choi et al., 2020). Even though all patients in this study have not complained unexpected symptoms or signs, there is a possibility for unintended efficacy on the cholinergic system for better or worse; the long-term use of CA should be monitored with precaution.

In conclusion, to enrich the listening comprehension in older adults with ARHL, it is necessary to increase audibility and to improve other factors associated with central auditory processing or cognition by using approaches from multiple perspectives. CA could be an affordable therapeutic option for older adult patients with ARHL showing poor WRS.

## Data Availability Statement

The original contributions presented in the study are included in the article/Supplementary Material, further inquiries can be directed to the corresponding author/s.

## Ethics Statement

The studies involving human participants were reviewed and approved by the Institutional Review Board (IRB) of Severance Hospital (4-2017 1152). The patients/participants provided their written informed consent to participate in this study.

## Author Contributions

GN and JJ: conceptualization and formal analysis. GN: methodology, resources, and writing—original draft preparation. JJ: investigation. GN, SK, JK, HN, SJ, and YS: data curation. JJ: writing—review and editing, visualization, supervision, project administration, and funding acquisition. All authors contributed to the article and approved the submitted version.

## Conflict of Interest

The authors declare that the research was conducted in the absence of any commercial or financial relationships that could be construed as a potential conflict of interest.
