# Corrigendum: Supplementary Effect of Choline Alfoscerate on Speech Recognition in Patients With Age-Related Hearing Loss: A Prospective Study in 34 Patients (57 Ears)

**DOI:** 10.3389/fnagi.2021.730527

**Published:** 2021-07-23

**Authors:** Gina Na, Sang Hyun Kwak, Seung Hyun Jang, Hye Eun Noh, Jungghi Kim, SeungJoon Yang, Jinsei Jung

**Affiliations:** ^1^Department of Otorhinolaryngology, Yonsei University College of Medicine, Seoul, South Korea; ^2^Department of Otorhinolaryngology, Ilsan Paik Hospital, Inje University College of Medicine, Goyang, South Korea; ^3^Department of Otorhinolaryngology, St. Vincent Hospital, College of Medicine, The Catholic University of Korea, Seoul, South Korea

**Keywords:** age-related hearing loss, hearing aids, choline alfoscerate, abbreviated profile of hearing aid benefit, listening comprehension

In the original article, there was an error in the Funding statement. The correct number for the National Research Foundation of Korea (NRF), funded by the Ministry of Science, ICT & Future Planning is **NRF-2019R1A2C1084033**. The corrected Funding statement is shown below.

This work was supported by the National Research Foundation of Korea (NRF), funded by the Ministry of Science, ICT & Future Planning (grant number **NRF-2019R1A2C1084033** to JJ).

The authors apologize for this error and state that this does not change the scientific conclusions of the article in any way. The original article has been updated.

## Publisher's Note

All claims expressed in this article are solely those of the authors and do not necessarily represent those of their affiliated organizations, or those of the publisher, the editors and the reviewers. Any product that may be evaluated in this article, or claim that may be made by its manufacturer, is not guaranteed or endorsed by the publisher.

