# Supplementary material for: Supplementary Effect of Choline Alfoscerate on Speech Recognition in Patients With Age-Related Hearing Loss: A Prospective Study in 34 Patients (57 Ears)
Source: Front Aging Neurosci. 2021 Jun 4;13:684519. doi: 10.3389/fnagi.2021.684519 (PMC8211767; doi:10.3389/fnagi.2021.684519)
Supplement: Supplementary file 1 [file Table_1.DOCX]

Supplementary Material

# Supplementary Figures and Tables

## Supplementary Figures

**
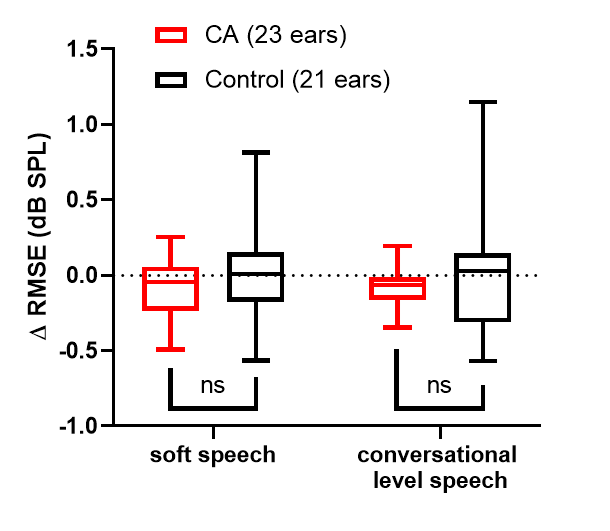
**

**Supplementary Figure 1.** Changes in root mean square error (RMSE) of fitting proximity. At 1 month and 1 year after wearing hearing aid, there was no significant between-group difference in the RMSE, calculated from the targeted gain (NAL-NL2) and aided gain in rear-ear measurement, at the input sound of soft speech and conversational level speech.

**Supplementary Figure 2**. Audiological baseline assessment. (A) There was no between-group difference in the initial average pure tone audiogram without using hearing aids at each frequency. (B): The distribution of the baseline word recognition score as a function of the high-frequency threshold (PTA3, average at 1000, 2000, and 4000 Hz) without hearing aids. There were 9 (39.1%) and 4 (11.8%) ears in the choline alfoscerate (CA) and control groups, respectively, beneath the estimated border of hearing loss possibly resulting from the retro-cochlear lesion

**Supplementary Figure 3.** Abbreviated Profile of Hearing Aid Benefit scores after 1 month of using hearing aids. There was no significant between-group difference in the global and aversiveness scores (p = 0.34 and 0.65, respectively).

**Supplementary Table 1. Hearing aid fitting data**

|  | **CA group** | **Control group** | ***p*-value^†^** |
| --- | --- | --- | --- |
| **1 month** | **n = 14, 23 ears** | **n = 12, 21 ears** |  |
| REAR at 55 d BSPL, mean [SD], dB SPL | 62.74 [5.07] | 61.36 [6.63] | 0.44 |
| REAR at 65 dB SPL, mean [SD], dB SPL | 67.78 [4.78] | 66.56 [6.62] | 0.48 |
| **1 year** | **n = 14, 23 ears** | **n = 20, 34 ears** |  |
| REAR at 55 dB SPL, mean [SD], dB SPL | 65.99 [6.88] | 64.78 [8.48] | 0.57 |
| REAR at 65 dB SPL, mean [SD], dB SPL | 77.04 [5.23] | 77.81 [7.06] | 0.66 |

Note: CA, choline alfoscerate; REAR, rear ear aided response; ^†^ Independent t-test; ^‡^ Mann-Whitney U
